# Model of Close Packing for Determination of the Major Characteristics of the Liquid Dispersions Components

**DOI:** 10.1155/2014/615236

**Published:** 2014-07-17

**Authors:** Kiril Hristov Kolikov, Dimo Donchev Hristozov, Radka Paskova Koleva, Georgi Aleksandrov Krustev

**Affiliations:** ^1^Department of Mathematics and Informatics, Plovdiv University “Paisii Hilendarski,” 24 Tsar Assen Street, 4000 Plovdiv, Bulgaria; ^2^Department of Physics, University of Food Technologies, 26 Maritsa Boulevard, 4000 Plovdiv, Bulgaria; ^3^Department of Physics, Plovdiv University “Paisii Hilendarski,” 24 Tsar Assen Street, 4000 Plovdiv, Bulgaria

## Abstract

We introduce a close packing model of the particles from the disperse phase of a liquid dispersion. With this model, we find the sediment volumes, the emergent, and the bound dispersion medium. We formulate a new approach for determining the equivalent radii of the particles from the sediment and the emergent (different from the Stokes method). We also describe an easy manner to apply algebraic method for determining the average volumetric mass densities of the ultimate sediment and emergent, as well as the free dispersion medium (without using any pycnometers or densitometers). The masses of the different components and the density of the dispersion phase in the investigated liquid dispersion are also determined by means of the established densities. We introduce for the first time a dimensionless scale for numeric characterization and therefore an index for predicting the sedimentation stability of liquid dispersions in case of straight and/or reverse sedimentation. We also find the quantity of the pure substance (without pouring out or drying) in the dispersion phase of the liquid dispersions.

## 1. Introduction

The volumetric mass density is an important variable, which participates in determining the quality of the liquid dispersions, as discussed elsewhere [1, 2].

The liquid dispersions (LDs) consist of dispersion medium (most often water) and dispersion phase. In the dispersion phase, the LDs there are particles with larger and particles with smaller volumetric mass density than the average density of the dispersion medium, which we shall call* heavy particles *and* light particles*. The heavy particles, in the conditions of homogeneous gravitational and centrifugal field, are displaced downwards (in direction of the field intensity vector) in containers with LD, and this phenomenon is called* straight sedimentation *or settling. The light particles are displaced upwards in containers with LD, and this phenomenon is called* reverse sedimentation (creaming) *or emergence. In the suspensions there are more heavy particles, and in the emulsions there are light particles. After reaching the condition of complete phase separation of the LD, we have ultimate (maximal) sediment and emergent.

The scientific studies of the LD are developed mainly in two directions:

1. determining the size and the density of the particles in the dispersion phase of LD and their distribution according to size and analyzing the process of their settling, that is,* sedimentation analysis *as discussed by Hodakov and Udkin [3];
2. studying the ability of LD, in unchanged external impacts, to maintain for definite time the homogeneous distribution of the dispersion phase in the dispersion medium, that is, studying the* sedimentation stability *as discussed by Hodakov and Udkin [3].

For sedimentation analysis and determining the sedimentation stability of LD, there are widely used visual and optical methods and devices, including taking samples from the different levels of the volume of the tested LD as discussed by Westwood and Kabadi [4], measuring the mass of the sedimented particles, and applying electricity or some radiation as discussed by Püttmer et al. [5]. The optical methods can achieve a very high degree of sensitivity and automation, but they have a serious disadvantage—the tested LD must be transparent for the applied length waves. Therefore, the tested sample is often diluted. The dilution, however, changes the properties of the LD! This problem is avoided by using the liquid separated by centrifugation. Moreover, the optical devices (spectrophotometers) have expensive equipment.

In this paper, we introduce a model of close packing of the particles from the dispersion phase of the LD. With this model, we determine the* volume *of the sediment and the emergent of the LD.

The empirical formula of Stokes for finding the radius of the equivalent spherical particles from the dispersion phase is well known as discussed by Hodakov and Udkin [3]. We formulate a new, much simpler approach for finding the average* Stokes radii* of the particles from the dispersion phase. We also offer a simple algebraic method for determination, under given temperature and external pressure, of the* average volumetric mass density* of the LD components—of the maximum sediment and the maximum emergent, as well as the dispersion medium, without using pycnometers as discussed by Westwood and Kabadi [4] or densitometers as discussed by Püttmer et al. [5]. With the determined densities, we find the components* masses* and also the volumetric density of the dispersion phase of the tested LD.

Based on the densities of the components we defined, for the first time, also a dimensionless scale—numeric characteristic for characterizing the quality, therefore, for predicting the sedimentation stability of liquid dispersions.

There is not a physical theory, yet, for the quantitative determination of the dispersion phase and the dispersion medium of LD. The most used method is drying, which is a long process, resulting also in the destruction of the tested substance.

Through the mass of the LD, the densities, and the linear sizes of its components, we determine the* percentage of the pure substance* in the dispersion phase, as well as the percentages of the bound and the free dispersion medium of LD.

All these results are obtained without complex methods and devices. We use straight prismatic or cylindrical transparent containers, where we pour homogenized LD. The full containers are placed under the influence of gravitational or centrifugal filed under constant temperature and external pressure.

We assume that the size of the container is large enough, so the wall-adjacent effect can be avoided.

## 2. Model of Close Packing of the Particles from the Dispersion Phase of Liquid Dispersion

We shall call the distribution of the particles from the dispersion phase, assumed to be spherical and touching adjacent particles, after the complete separation phase,* a model of the close packing of the dispersion phase.*

**Figure 1:**
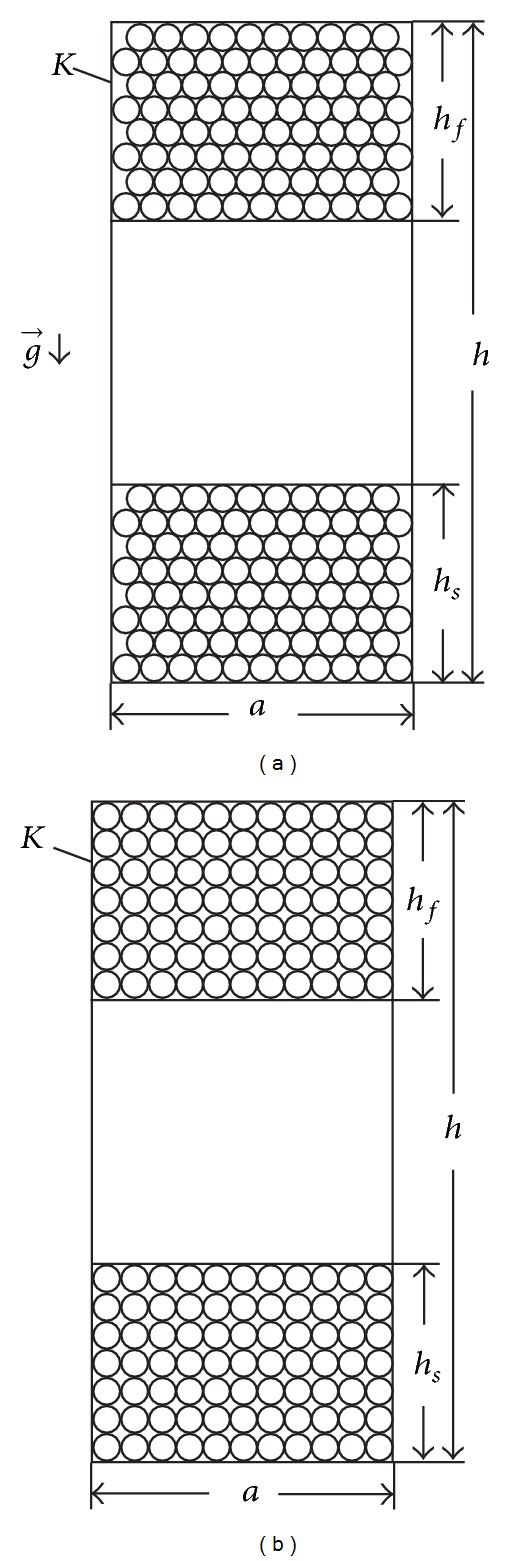
Vertical axial section of straight square prismatic container with separated components of the liquid dispersion: (a) model of perfect close packing of the particles from the dispersion phase; (b) model of nonperfect close packing of the particles from the dispersion phase.

Figure 1 shows axial section of straight containers *K*, which we assume have square foundations.

We assume that at the initial moment in the container homogenized LD is poured and that it is placed in conditions of static homogeneous gravitational or centrifugal filed under constant temperature and external pressure. In the case of centrifugal filed, because of the relatively small sizes of *K*, compared with those of a centrifuge, we can assume that the centrifugal filed in *K* is practically homogeneous.

After the phase separation of LDs, sharp macroscopic flat border is formed between the components of the dispersion phase, with separated dispersion medium in *K*.

In the model of the close packing of the particles from the dispersion phase offered by us, it is assumed that their shape is slightly different from spherical particles, which for most of the LD is satisfactorily met as discussed by Hodakov and Udkin [3]. Moreover, as a result of the homogenizing of the LD and the conditions, in which the sedimentation takes place, we can also assume sequential particle distribution along the horizontals in the maximum sediment and emergent.

The concentration gradient of the dispersion particles during the formation of the sediment and the emergent also significantly contributes to this distribution along the horizontals. (As end result, this gradient becomes 0.)

After the ultimate separation, a part of the dispersion medium is located among the particles from the dispersion phase of the sediment and the emergent. This dispersion medium (in most cases, water) is called* bound (nonfree)*. The dispersion medium, separated outside the sediment and the emergent, is called* unbound (free)*. We shall emphasize that unlike the free dispersion medium, the bound dispersion medium cannot be separated even during centrifugation, because between the particles of the medium and the particles of the dispersion phase there are huge quantum-mechanical (close) interactions—much larger than any centrifugal.

It should be taken into account that by approaching the surfaces of two particles the forces of attraction between them grow relatively slow—for dispersion interactions by the law 1/*τ* ^3^, where *τ* is the distance between the particles. Contrariwise, the forces of repulsion are practically insignificant to enough short distance, but, by further reduction of *τ*, these forces are increasing rapidly, by the low 1/*τ* ^*n*^, where *n* ≈ 10. As a result, by approaching the surfaces of the particles to equilibrium state of a close packing, the majority of the work is for the forces of attraction and the depth of the minimum of the potential curve is close in absolute value to the work of the forces of attraction, that is, molecular component of the energy. Furthermore, the energy and the interaction force between the particles are determined not only by the distance between the particles and the value of the complex constant of Hamaker, but also by the size and the shape of the interacting particles discussed by Shchukin et al. [6].

Let the square of the basis *K* have an internal side length *a* and let the height of the LD in *K* be *h*. With *h* we designate the height of the ultimate sediment, and with *h*_*f*_ we designate the height of the ultimate emergent (Figures 1(a) and 1(b)).

We shall find the number of the particles from the dispersion phase. We assume that an ultimate (maximal) sediment (emergent) in close packing, along the length *a*, can be applied *i* number of dispersion particles and each of these particles has an average Stokes radius *r* = *r*_*s*_  (*r* = *r*_*f*_). We also assume that the height *h* = *h*_*s*_  (*h* = *h*_*f*_) can be applied *j* number of such particles.

- Considering the model of perfect close packing of the particles from the dispersion phase, due to the large values of *i* and *j* we can ignore the difference in one particle through row or column.

Then, the number of the particles in the sediment (emergent) is *n* = *i* ^2^ *j*. Moreover, because of simple geometrical considerations it follows that $h=\left(j-1\right)\sqrt{3}+2r=j\sqrt{3}r+\left(2-\sqrt{3}\right)r$.

Due to the large values of numbers *j* and *r* ≪ *j* we can assume that $h=j\sqrt{3}r$; that is,

(1) $$\begin{array}{c} a=2ir;\;\;h=j\sqrt{3}r. \end{array}$$

(b) Considering the model of nonperfect close packing of the particles from the dispersion phase,
  (2) $$\begin{array}{c} a=2ir;\;\;h=2jr. \end{array}$$

Therefore, we assume the average values of *a* and *h*; that is,

(3) $$\begin{array}{c} a=2ir;\;\;h=\left(1+\frac{\sqrt{3}}{2}\right)jr. \end{array}$$

## 3. Volume of the Sediment and the Emergent in Liquid Dispersion

According to formula (3) for the volume *V* = *V*_*s*_  (*V* = *V*_*f*_) of the full ultimate sediment (emergent) in *K*, we have $V=a^{2}h=2i^{2}j\;\left(2+\sqrt{3}\right)r^{3}$, where *r* is the radius of the Stokes particles of the dispersion phase.

On the other hand, *V* = *V*′ + *V*′′, where *V*′ is the* volume of the dispersion phase* in this sediment (emergent) and *V*′′ is the volume of the* bound dispersion medium* of the sediment (emergent). The following equation is valid:

(4) $$\begin{array}{c} V^{\prime }=\frac{4}{3}\pi \;r^{3}i^{2}j. \end{array}$$

Therefore, $V^{\prime \prime }=V-V^{\prime }=2i^{2}j\left(2+\sqrt{3}-(2/3)\pi \right)r^{3}$. From the obtained expressions for *V*, *V*′, and *V*′′ we determine that

(5) V′V=2π3(2+3)≈0,56,V′′V=1−2π3(2+3)≈0,44.

Then,

(6) $$\begin{array}{c} V^{\prime }\approx 0,56a^{2}h;\;\;V^{\prime \prime }\approx 0,44a^{2}h. \end{array}$$

From the equations in (6), it follows that *V*′ ≈ 1,3*V*′′.

Let *ρ*′ = *ρ*_*s*_′  (*ρ*′ = *ρ*_*f*_′) be the average volumetric mass density of the particles from the dispersion phase in the ultimate sediment (emergent). In addition, let *ρ*′′ be the average density of the dispersion medium (bound or free) in the LD. Then, according to formulae (6), for the masses *m*′ and *m*′′, respectively, in the dispersion phase and the bound dispersion medium in the ultimate sediment/emergent in *K*, we have

(7) $$\begin{array}{c} m^{\prime }=V^{\prime }\rho^{\prime }\approx 0,56a^{2}h\rho^{\prime };\;\;m^{\prime \prime }=V^{\prime \prime }\rho^{\prime \prime }\approx 0,44a^{2}h\rho^{\prime \prime }, \end{array}$$

where *h* = *h*_*s*_(*h* = *h*_*f*_).

In (7), if *ρ*′ = *ρ*_*s*_′, then *ρ*′ > *ρ*′′, and if *ρ*′ = *ρ*_*f*_′, then *ρ*′ < *ρ*′′.

In our studies we obtain approximated formulas in which the practically negligible distance between the particles is ignored; that is, we assume that the particles of the dispersion phase by ultimate sedimentation are closely fitted to each other. This does not affect the calculations, because we do not consider only the case when the particles are perfectly arranged closely to each other.

## 4. Determination of the Average Stokes Radii of the Particles in the Sediment and in the Emergent

Using (3), we can find the numbers *i* = *i*_*s*_  (*i* = *i*_*f*_) or *j* = *j*_*s*_  (*j* = *j*_*f*_) and hence the average Stokes radius of the particles from the sediment (emergent). To this end, we count the dispersion particles symmetrically around the center of the masses of the ultimate sediment (emergent), along the vertical in a chosen linear unit of distance. (This is done, because even in monodisperse system the dispersion particles are not of absolutely the same size.) Then the obtained number is multiplied by the number of single distances, contained in *h*. This is how we find *j*, and then according to (3),

(8) $$\begin{array}{c} i=\frac{a\left(1+\left(\sqrt{3}/2\right)\right)}{2h}j. \end{array}$$

Hence, from (3) for the average Stokes radii of the particles from the sediment or the emergent, we have

(9) $$\begin{array}{c} r=\frac{h}{\left(1+\left(\sqrt{3}/2\right)\right)j}=\frac{a}{2i}. \end{array}$$

If the LD in *K* is polydisperse, the obtained radii *r* of the particles from the sediment or the emergent are determined in average values.

With this, we offer a new method, different from Stokes', for determination of the average Stokes radii in dispersion particles in LD. The determination of the *n* = *i* ^2^ *j* of the dispersion particles from the ultimate sediment or emergent in LD in *K* provides important information also for the food, medical, chemical, and so forth industries.

## 5. Algebraic Method for Determining the Average Densities and the Masses of the Components of a Liquid Dispersion

The densities of the ultimate sediment and emergent, as well as the free dispersion medium, can be defined with the widely used* pycnometers* as discussed by Westwood and Kabadi [4]. But to this end, these three components of the LD must be mechanically separated. This requires the ultimate emergent to be removed from the container first and then pouring out the free dispersion medium and finally there is access to the ultimate sediment. After that, the three substances samples are taken, which are placed in the pycnometer. The pycnometer method for measuring the densities is easy to apply and inexpensive, but the measurement takes a lot of time.

The measurement can be also done in a noncontact way, by means of* ultrasound densitometers* as discussed by Püttmer et al. [5]. These devices, however, use complex equipment and are expensive.

**Figure 2:**
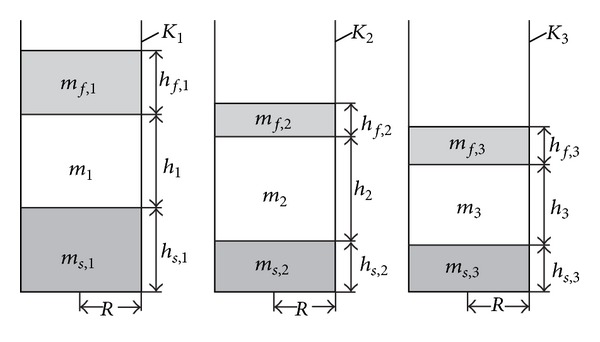
Vertical axial section of three equal straight cylindrical containers with separated components of liquid dispersions.

We offer an* algebraic method*, with significantly simplified measurements. It employs containers—prismatic or cylindrical. At the beginning, the masses of the LD must be measured, which need to be equal, but taken in different proportions from the dispersion phase. The LD is placed in the respective containers under constant temperature and external pressure. After the formation of the ultimate sediment and emergent and the free dispersion medium between them, in the conditions of gravitational or centrifugal field, we perform elementary linear measurements of the geometrical sizes of the separated particles in the containers with LD (Figure 2). The densities and the masses of the components are determined with simple algebraic operations.

As it is not necessary to count the particles in the ultimate sediment and emergent, in this case we can use straight cylindrical containers. Let *K*_*i*_  (*i* = 1,2, 3) be such containers, which for convenience we assume are same, with radii of the main planes of *r*, as they are presented in the vertical axial sections in Figure 2. The three containers contain LD with the same components of the dispersion medium and the dispersion phase, taken in different proportions. (These proportions can be achieved, for example, by minimal dilution with dispersion medium of the given LD, that is, by increasing the relative share of the dispersion medium in the different containers.)

Let us assume that at the initial moment *t* = 0 the tested LD is in homogenized condition; that is, the dispersion phase of this dispersion is evenly distributed in the whole volume of the containers. Moreover, the containers are placed under specific constant temperature and external pressure.

In the conditions of gravitational or centrifugal field comes a moment of time *t* > 0, when in LD of *K*_*i*_  (*i* = 1,2, 3) appears a condition of ultimate sediment and emergent with constant heights of their volumes. Thus within time *t* complete phase separation is achieved; that is, the volumes and the masses of the formed ultimate sediment and ultimate emergent in *K*_*i*_ (Figure 2) no longer change—the heavy particles have settled, and the light particles have emerged.

Some cases of LD are possible, when free dispersion medium and only ultimate sediment or only ultimate emergent are formed. Some more complicated cases are also possible, when more than one sediment and/or emergent are formed.

The values of the average density of the substance in the ultimate sediment *ρ*_*s*_ of the average density of the substance in the ultimate emergent *ρ*_*f*_ and the density of the dispersion medium *ρ* = *ρ*′′ in the containers *K*_*i*_  (*i* = 1,2, 3) are the same.

Characteristics of the ultimate sediment and emergent are the lights of their heights *h*_*s*,*i*_ and *h*_*f*,*i*_  (*i* = 1,2, 3) in *K*_*i*_, which are constant after certain moment of time *t*. With *h*_*i*_  (*i* = 1,2, 3) we designate the heights of the free dispersion medium, and with *m*_*s*,*i*_, *m*_*f*,*i*_, *m*_*i*_ (*i* = 1,2, 3) we designate the masses of the ultimate sediment and emergent and the free dispersion medium of LD in *K*_*i*_ from Figure 2. Then we can write

(10) ms,i=πR2hs,iρs, mf,i=πR2hf,iρf, mi=πR2hiρ,(i=1,2,3),

where *R* is the radius of the internal bases of the containers. With *M*_*i*_  (*i* = 1,2, 3) we designate the total masses of LD, which fill up *K*_*i*_. We consider these masses to be known (measured). According to the law on conservation of masses, we have the following system of three linear equations:

(11) $$\begin{array}{c} m_{s,i}+m_{f,i}+m_{0,i}=M_{i},\;\;i=1,2,3. \end{array}$$

If *H*_*i*_ = *h*_*s*,*i*_ + *h*_*i*_ + *h*_*f*,*i*_, and ${\overset{-}{\rho }}_{i}$ is the average volumetric mass density of LD in *V*_*i*_, then it is known that $k_{i}=M_{i}/\pi R^{2}=H_{i}{\overset{-}{\rho }}_{i}\;\;(i=1,2,3)$. Then from (10) and (11) the system follows:

(12) $$\begin{array}{c} h_{s,i}\rho_{s}+h_{f,i}\rho_{f}+h_{i}\rho =k_{i},\;i=1,2,3. \end{array}$$

By solving (12), the three densities are determined—of the ultimate sediment, of the ultimate emergent, and of the dispersion medium:

(13) $$\begin{array}{c} \rho_{s}=\frac{D_{1}}{D},\;\;\;\;\;\rho_{f}=\frac{D_{2}}{D},\;\;\;\;\rho =\frac{D_{3}}{D}, \end{array}$$

where *D*, *D*_1_, *D*_2_, and *D*_3_, according to the Cramers rule, are determinants of third order.

Since the proportions of the components in LD are different, then the equations in the system (11) are not equivalent and the system (12) is consistent. But, if we pour the same LD, even in three different in size and shape straight containers, then the equations in (11) will be proportional. Then the system (12) will be inconsistent.

From (10) and (13), we can determine the masses *m*_*s*,*i*_, *m*_*f*,*i*_, *m*_*i*_, as well as the mass *m*_*p*,*i*_ = *m*_*s*,*i*_ + *m*_*f*,*i*_ of the dispersion phase, for any *i*. Then we also find the density *ρ*_*p*_ of the dispersion phase in the tested LD:

(14) $$\begin{array}{c} \rho_{p}=\frac{h_{s,i}\rho_{s}+h_{f,i}\rho_{f}}{h_{s,i}+h_{f,i}}. \end{array}$$

If we need to determine only the density *ρ*_*p*_ of the dispersion phase and the density *ρ* of the dispersion medium, two containers *K*_1_ and *K*_2_ are enough and a system with two linear equations

(15) $$\begin{array}{c} m_{p,i}+m_{1}=M_{1}⟺\left(h_{s,i}+h_{f,i}\right)\rho_{p}+h_{i}\rho =k_{i},\;i=1,2. \end{array}$$

Thus

(16) $$\begin{array}{c} \rho_{p}=\frac{D_{1}}{D},\;\;\rho =\frac{D_{2}}{D}, \end{array}$$

where *D*, *D*_1_, and *D*_2_, according to the Cramers rule, are determinants of second order.

If we know the density of one of the ingredients, for example, *ρ* for distilled water, then also two containers *K*_1_ and *K*_2_ are enough. Then *ρ*_*s*_ and *ρ*_*f*_ are also determined with a system with two linear equations

(17) ms,i+mf,i+mi=Mi⟺hs,iρs+hf,iρf=ki−hiρ,i=1,2,

which is solved analogically to system (12).

In case when the density *ρ* of the dispersion medium is known and we have only ultimate sediment or ultimate emergent, it is sufficient to use only one container *K* = *K*_1_. Then we have only one linear equation

(18) $$\begin{array}{c} m_{s}+m=M\;\text{or}\;\;m_{f}+m=M; \end{array}$$

that is,

(19) $$\begin{array}{c} \rho_{p}=\frac{k-h\rho }{h_{s}}\;\text{or}\;\;\rho_{p}=\frac{k-h\rho }{h_{f}}, \end{array}$$

where *ρ*_*p*_ = *ρ*_*s*_ or *ρ*_*p*_ = *ρ*_*f*_ and *k* = *M*/*πR* ^2^.

Because *m*_*s*_ = *m*′ + *m*′′  (*m*_*f*_ = *m*′ + *m*′′), where *m*′ is the mass of the dispersion phase and *m*′′ is the bound dispersion medium in the ultimate sediment (emergent), and according to formula (19), we can find the density *ρ*′ of the dispersion phase

(20) $$\begin{array}{c} \rho^{\prime }=\frac{\rho_{p}-0,44\rho }{0,56}. \end{array}$$

In this case, when the tested LD has sediments and emergents, consisting of several components, then the density of each component can be determined through a measurement with more containers. In the general case, we use a system consisting of *n* linear equations and *n* unknowns, which is present in the following way:

(21) $$\begin{array}{c} \overset{p}{\underset{k=0}{\sum }}m_{s_{k},i}+\overset{l}{\underset{j=0}{\sum }}m_{f_{j},i}+m_{i}=M_{i},\;i=1,2,...,n, \end{array}$$

where *n* = *p* + *l* + 1. From (21) the unknown densities *ρ*_*s*_*k*_,*i*_, *ρ*_*f*_*j*_,*i*_, and *ρ* are determined.

From (13), (14), (16), and (19), using the method shown in Kolikov et al. [7], we can determined the maximum absolute inaccuracies (errors) Δ*ρ*_*s*_, Δ*ρ*_*f*_, Δ*ρ*_*p*_, and Δ*ρ* and the maximum relative inaccuracies (errors) Δ*ρ*_*s*_/*ρ*_*s*_, Δ*ρ*_*f*_/*ρ*_*f*_, Δ*ρ*_*p*_/*ρ*_*p*_, and Δ*ρ*/*ρ* discussed by Kolikov et al. [8].

## 6. Characterization Scale for Sedimentation Stability

There are different methods for evaluation of the sedimentation stability *S* of LD. One of the most popular methods is the* visual method*. The visual method measures the observed displacement over a period of time of the horizontal separation line between the clear and the muddy part of the tested LD as discussed elsewhere [9, 10]. The method employs transparent container and a clock.

From the* instrumental methods*, most popular ones in determining the sedimentation stability are the optical absorption methods—through measuring the intensity of the light passing through the LD as discussed elsewhere [11, 12].

According to our method, the evaluation of *S* is done through the densities of the components of the LD and it is not necessary to measure the time, and optical transmission of LD is no required. Moreover, we introduce* scale for characterization of the sedimentation stability S*!

Let one particle of the dispersion phase with volume *V* and density *ρ*_*p*_ be located in dispersion medium with density *ρ*, in the conditions of gravitational field. Then the value of the resultant force of its weight and the Archimedean force is

(22) $$\begin{array}{c} F=Vg\left(\rho_{p}-\rho \right), \end{array}$$

where *g* is gravitational constant. (In (22) we neglect the friction force, which is very little for small velocities of the particle.)

Therefore, if the particle is heavy; that is, *ρ*_*p*_ > *ρ*⇔*ρ*/*ρ*_*p*_ < 1, then *F* > 0 and we have straight sedimentation (settling). And if the particle is light; that is, *ρ*_*p*_ < *ρ* ⇔ *ρ*_*p*_/*ρ* < 1, then *F* < 0 and we have reverse sedimentation (emergence).

Thus we suggest the evaluation of the sedimentation stability *S* to be done through the following dimensionless physical variables: *I*_*s*_ = *ρ*/*ρ*_*s*_, *I*_*f*_ = *ρ*_*f*_/*ρ*  (*ρ*_*s*_, *ρ* ≠ 0) and hence through the variable

(23) $$\begin{array}{c} I=\max\left(I_{s},\;I_{f}\right)=\max\left(\frac{\rho }{\rho_{s}},\;\frac{\rho_{f}}{\rho }\right). \end{array}$$

The number *I* is the* sedimentation stability index* in a dimensionless scale for characterization of *S*. Since 0 < *I*_*s*_, *I*_*f*_ ≤ 1, then 0 < *I* ≤ 1, that is, the scale is the interval (0,1]. The more the index *I* of an LD is closer to 1, the more stable the LD is, and the closer the index *I* is to 0, the more unstable the LD is. Thus, if *I* = 1, the LD is absolutely sedimentary stable; that is, there is no phase separation.

We shall notice that if the sediment is absent in the LD, then *I* = *I*_*f*_ ∈ (0,1], and if the emergent is absent, then *I* = *I*_*s*_ ∈ (0,1].

## 7. Determining the Percentage of the Substance in the Sediment and the Emergent

The amount of the substance in the dispersion phase is a major factor, which determines the quality of many products. The drying is widely used method for testing the dry matter in different suspensions. To this end, a sample is taken from the homogenized LD, which is placed in a suitable container, weighted with a scale, and placed in a drier for drying until constant weight is obtained. This weight is divided into the initial (not dried) full weights of the sample and then multiplied by 100. The obtained value is called* dry substance percentage *of LD. The disadvantages of this method are its relatively long duration and the spending of significant thermal energy for drying a unit of weight from the tested substance. Moreover, the heating, usually to 105°C, required by the standard, results in destruction of the treated substance, and it can only be used for suspensions.

We offer a different method, which does not have these flaws.

If *K* is a straight cylindrical container, filled-up with LD with weight *M* and internal radius of the bases *R*, then, according to formula (7), the masses of the dispersion phase and the bound dispersion medium in the ultimate sediment or emergent are respectively *m*′ = 0,56*πR* ^2^ *hρ*′, and *m*′′ = 0,44*πR* ^2^ *hρ*′′. Then, knowing the geometrical parameters of the used container, as well as the heights, masses, and densities, we have in percentage the amount of the substance *P*′ in the ultimate sediment or emergent and of the bound dispersion medium *P*′′, respectively:

(24) $$\begin{array}{c} P^{\prime }=\left(\frac{m^{\prime }}{M}\cdot 100\right)\%=\left(\frac{0,56\pi R^{2}h\rho^{\prime }}{M}\cdot 100\right)\%, \end{array}$$

(25) $$\begin{array}{c} P^{\prime \prime }=\left(\frac{m^{\prime \prime }}{M}\cdot 100\right)\%=\left(\frac{0,44\pi R^{2}h\rho^{\prime \prime }\;}{M}\cdot 100\right)\%. \end{array}$$

The percentage *P* of the free dispersion medium can also be calculated with our method; namely

(26) $$\begin{array}{c} P=\left(\frac{\pi R^{2}\left(H-h_{s}-h_{f}\right)\rho^{\prime \prime }\;}{M}\cdot 100\right)\%. \end{array}$$

Let us denote that unlike the method of drying until constant weight, through formula (24), the percentage *P*′ of the tested substance is determined with the participation of the bound dispersion medium. We take into account only the amount of the pure dispersion phase in the specific sediment or emergent. Through drying, complete dryness of the sample cannot be achieved in practice—in the sediment, there always remains some percentage bound dispersion medium. Therefore, we cannot expect to have coinciding percentage of dry substance, determined according to the two methods.

Let us consider a case of sediment. If ${\overset{-}{P}}_{s}^{\prime }$ is the percentage of dry substance in the sediment, determined with the drying method, then, for practical reasons, we can write down modification of formula (24); namely,

(27) $$\begin{array}{c} {\overset{-}{P}}_{s}^{\prime }=\varepsilon P_{s}^{\prime }\approx \left(\varepsilon \frac{0,56\pi R^{2}h_{s}\rho^{\prime }\;}{M}\cdot 100\right)\%. \end{array}$$

Here the variable *ε* is a conversion factor, which can change within the limits 1 < *ε* < (*m*_*s*_′ + *m*_*s*_′′)/*m*_*s*_′, where *m*_*s*_′ + *m*_*s*_′′ = *V*_*s*_′*ρ*_*s*_′ + *V*_*s*_′′*ρ*′′ is the total weight of the sediment (dispersion phase and bound dispersion medium). The unobtainable value *ε* = 1 corresponds to the dry substance in the ultimate sediment in case of complete drying of the sample in *K*, that is, without bound dispersion medium, as with formula (24) we offer. If the ultimate sediment is not subjected to drying, then the value *ε* = (*m*_*s*_′ + *m*_*s*_′′)/*m*_*s*_′ ≈ 1 + (*ρ*′′/*ρ*_*s*_′) < 2. Thus for the conversion factor in formula (27) we have 1 < *ε* < 2.

## 8. Example

We present models of two dispersion system suspensions from calcium carbonate powder, respectively, 7,00 g and 11,00 g, measured with electronic scale with accuracy of 0,01 g and distilled water, respectively, 50 mL and 65 mL, measured with graduated cylinders with accuracy of 0,5 mL. In order to evaluate the accuracy of our method, we assume the densities of CaCO_3_ and the distilled H_2_O to be unknown.

**Figure 3:**
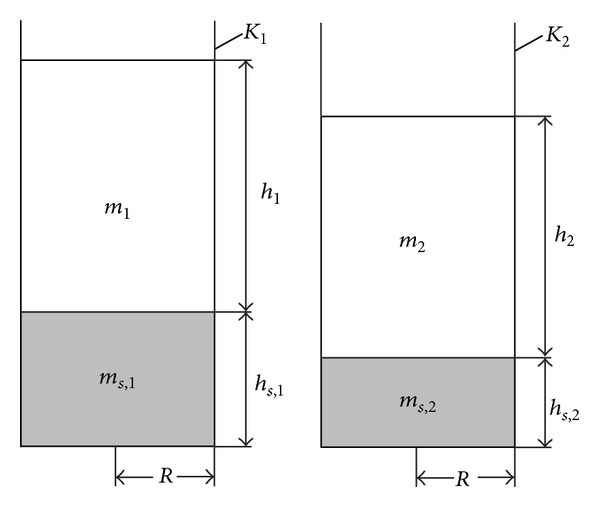
Vertical axial sections of two identical straight cylindrical containers with ultimate sedimentation.

In order to determine the densities, respectively, *ρ*_*s*_ of the ultimate sediment and *ρ* of the dispersion medium, we use two identical cylindrical containers, *K*_1_ and *K*_2_, with radius of the internal basis *R* = 0,013 m (Figure 3).

In the two containers we pour the two homogenized suspensions (their components are in different proportions). We perform 6 observations with room temperature 20°C.

Table 1 presents the values of the measured variables *R*, *h*_*s*,1_, *h*_1_, *M*_1_ (for container *K*_1_) and *R*, *h*_*s*,2_, *h*_2_, *M*_2_ (for container *K*_2_) during the 6 observations.

For densities *ρ*_*s*_ and *ρ*, according to formula (15), we find with the system of linear equations

(28) $$\begin{array}{c} |\begin{array}{c} m_{s,1}+m_{1}=M_{1}\\ m_{s,2}+m_{2}=M_{2} \end{array}⟺|\begin{array}{c} h_{s,1}\rho_{s}+h_{1}\rho =\frac{M_{1}}{\pi R^{2}}\\ h_{s,2}\rho_{s}+h_{2}\rho =\frac{M_{2}}{\pi R^{2}} \end{array}. \end{array}$$

Its solutions are

(29) $$\begin{array}{c} \rho_{s}=\frac{D_{1}}{D}=\frac{1}{\pi R^{2}}\frac{M_{1}h_{2}-h_{1}M_{2}}{h_{s,1}h_{2}-h_{1}h_{s,2}},\\ \rho =\frac{D_{2}}{D}=\frac{1}{\pi R^{2}}\frac{h_{s,1}M_{2}-M_{1}h_{s,2}}{h_{s,1}h_{2}-h_{1}h_{s,2}}. \end{array}$$

Then for the densities of the ultimate sediment and the dispersion medium, we have, respectively, the average values *ρ*_*s*_ ≈ 1,70 g*·*cm^−3^, and *ρ* ≈ 1,03 g*·*cm^−3^, which are also presented in Table 1.

For the index of the sedimentation stability we find

(30) $$\begin{array}{c} I=\frac{\rho }{\rho_{s}}\approx 0,61. \end{array}$$

The value of *I* according to our scale (0,1] shows that the tested LD has a little over the average sedimentation stability.

According to the model discussed by Kolikov et al. [7], we shall determine the maximum absolute inaccuracies Δ*ρ*_*s*_ and Δ*ρ* for the established densities *ρ*_*s*_ and *ρ*, as well as the maximum relative inaccuracies Δ*ρ*_*s*_/*ρ*_*s*_ and Δ*ρ*/*ρ*. If Δ*M*_*i*_, Δ*h*_*i*_, Δ*h*_*s*,*i*_  (*i* = 1,2), and Δ*R* are absolute inaccuracies of *M*_*i*_, *h*_*i*_, *h*_*s*,*i*_, and *R*; then from (29) it follows that

(31) Δρs=1|D|(|h2πR2||ΔM1|+|h1πR2||ΔM2|+|D1Dhs,2−M2πR2||Δh1|+|M1πR2−D1Dhs,1||Δh2|+|D1Dh2||Δhs,1|+|D1Dh1||Δhs,2|+|2D1R||ΔR|),

(32) Δρ=1|D|(|hs,2πR2||ΔM1|+|hs,1πR2||ΔM2|+|D2Dhs,2||Δh1|+|D2Dhs,1||Δh2|+|M2πR2−D2Dh2||Δhs,1|+|D2Dh1−M1πR2||Δhs,2|+|2D2R||ΔR|),

where Δ*h*_1_ = 0,056 cm, Δ*h*_2_ = 0,067 cm, Δ*h*_*s*,1_ = Δ*h*_*s*,2_ = Δ*R* = 0 cm, Δ*M*_1_ = 0,27 g, and Δ*M*_2_ = 0,30 g.

Considering the calculations from Tables 2 and 3, we have the values of the maximum absolute inaccuracies Δ*ρ*_*s*_ ≈ 0,43 g*·*cm^−3^ and Δ*ρ* ≈ 0,18 g*·*cm^−3^.

From here we find the values of the maximum relative inaccuracies

(33) $$\begin{array}{c} \frac{\Delta \rho_{s}}{\rho_{s}}=\frac{0,43}{1,70}\approx 0,25,\;\;\frac{\Delta \rho }{\rho }=\frac{0,18}{1,03}\approx 0,17. \end{array}$$

Thus obtained value of *ρ*, at 20°C temperature, is within the error for the density of the distilled water, which shows the accuracy of our model.

Then we find the percentage *P*′ of the dry substance in the sediment and the percentages *P*′′ and *P* of the bound dispersion medium in the sediment and of the free dispersion medium, accordingly. We use formulae (20), (24), (25), and (26) and the data from Table 1. The results are shown in Table 4.

Because of the approximations in the calculations, the sum of the values of *P*′, *P*′′, and *P* is around 100%.

## 9. Discussion

Our model of close packing of the particles from the dispersion phase of the studied liquid dispersion offers a new method for finding the volumes and the masses of the sedimented substances in LD, as well as the average Stokes radii of the particles of these substances. Moreover, this study is distinguished with simplicity and its application is very cheap.

Our simple algebraic method offers an increased accuracy in determining the average densities and weights of the ultimate sediment and emergent, of the dispersion medium and of the dispersion phase in the liquid dispersion. The finding of these densities is achieved without the use of pycnometers or other more complex methods. This prevents the destructive effects in the LD, and the vibrations practically have no effect on the measurements results, unlike the methods existing until now.

Our method of determining the coefficient of the quantities of substances in the components of LD does not require pouring out the liquid from the container in order to measure the masses of the ultimate sediment and of the free dispersion medium (water). The known method of pouring out and drying is destructive. Using the drying method on LD, it is generally even impossible to determine the percentage of the substance amount in the emulsion. The method we propose overcomes this disadvantage.

A great advantage and particularly valuable for one method is that it has* numerical characteristic*—scale for evaluation of a given variable. The index *I* which we introduce in (23) is a dimensionless scale for characterization of the sedimentation stability *S*, naturally following from the law on the movement of the dispersion particle from the dispersion phase of LD. It provides a very easy way to evaluate *S* of one LD in straight and/or reverse sedimentation, through the densities *ρ*_*s*_, *ρ*_*f*_, and *ρ*, which are the solution of the system of linear equations of type (11).

The index *I* ∈ (0, 1] provides the possibility not only to evaluate and to predict the sedimentation stability, but also to compare different LDs according to their sedimentation stability. Moreover, the tests of the different LDs must be performed in constant temperature and external pressure.

## 10. Conclusion

The method, which we offer, does not require special equipment. The phase separation of LDs can be achieved in the conditions not only of gravitational (earth's), but also of centrifugal field. In the case of centrifugal field, the time interval *t* for determining the ultimate sediment and emergent can be much smaller, compared with time for treating the LD with the acceleration of gravity $\vec{g}$.

The advantage of our physicomathematical model for finding the Stokes radii in the dispersion phase is that it does not use complicated mathematical analysis for structuring the sedimentation curve.

The three variables *P*′, *P*′′, and *P*, characterizing in percentage the respective quantity of substance in the ultimate sediment or emergent, bound dispersion medium in the sediment, or the emergent and the free dispersion medium, are introduced in the literature for the first time with this paper.

And with the sedimentation stability index *I*, which we introduce, it is possible to demonstratively and without particular difficulties make qualitative analysis of suspensions and emulsions—products of the food, medical, cosmetic, chemical, and so forth industries. Such products are, for example, different suspensions and emulsions such as foods and drinks, medications and cosmetic products, paints and varnishes, and cement and lime solutions. This is particularly and technically useful in the creation of products with predetermined sedimentation stability.

## Figures and Tables

**Table 1:** Linear dimensions, masses, and densities of the components of liquid dispersions.

Exp.	*R* [cm]	*h*_*s*,1_ [cm]	*h*_1_ [cm]	*h*_*s*,2_ [cm]	*h*_2_ [cm]	*M*_1_ [g]	*M*_2_ [g]	*ρ*_*s*_ [g*·*cm^−3^]	*ρ* [g*·*cm^−3^]
1	1,3	2,4	6,3	3,6	7,6	56,07	73,97	1,69	1,03
2	1,3	2,4	6,1	3,6	7,6	55,17	74,33	1,73	1,02
3	1,3	2,4	6,1	3,5	7,8	55,09	74,39	1,72	1,02
4	1,3	2,4	6,2	3,6	7,7	55,74	74,95	1,74	1,02
5	1,3	2,4	6,2	3,6	7,7	55,51	74,58	1,71	1,02
6	1,3	2,4	6,2	3,6	7,6	55,57	73,83	1,63	1,06

Avg.	1,3	2,4	6,2	3,6	7,7	55,53	74,34	1,70	1,03

**Table 2:** Coefficient values of the absolute inaccuracies Δ*M*_1_, Δ*M*_2_, Δ*h*_1_, Δ*h*_2_, Δ*h*_*s*,1_, Δ*h*_*s*,2_, Δ*R*.

Exp.	|*D*|	|*D*_1_|	$\left|\frac{D_{1}}{D}\right|$	$\left|\frac{M_{1}}{\pi R^{2}}\right|$	$\left|\frac{M_{2}}{\pi R^{2}}\right|$	$\left|\frac{h_{1}}{\pi R^{2}}\right|$	$\left|\frac{h_{2}}{\pi R^{2}}\right|$	$\left|\frac{D_{1}}{D}h_{s,2}-\frac{M_{2}}{\pi R^{2}}\right|$	$\left|\frac{M_{1}}{\pi R^{2}}-\frac{D_{1}}{D}h_{s,1}\right|$	$\left|\frac{D_{1}}{D}h_{1}\right|$	$\left|\frac{D_{1}}{D}h_{2}\right|$	$\left|\frac{2D_{1}}{R}\right|$
1	4,44	7,5	1,69	10,56	13,93	1,19	1,43	7,85	6,5	10,65	12,84	11,54
2	3,72	6,44	1,73	10,39	14,00	1,15	1,43	7,77	6,24	10,55	13,15	9,90
3	2,63	4,57	1,74	10,37	14,01	1,15	1,47	7,92	6,19	10,61	13,57	7,04
4	3,84	6,63	1,73	10,50	14,11	1,17	1,45	7,88	6,35	10,73	13,32	10,20
5	3,84	6,64	1,73	10,45	14,05	1,17	1,45	7,82	6,30	10,73	13,32	10,22
6	4,08	6,61	1,62	10,47	13,90	1,17	1,43	8,07	6,58	10,04	12,31	6,28

Avg.	3,76	6,40	1,71	10,46	14,00	1,17	1,44	7,89	6,36	10,55	13,09	9,20

**Table 3:** Coefficient values of the absolute inaccuracies Δ*M*_1_, Δ*M*_2_, Δ*h*_1_, Δ*h*_2_, Δ*h*_*s*,1_, Δ*h*_*s*,2_, Δ*R*.

Exp.	|*D*|	|*D*_2_|	$\left|\frac{D_{2}}{D}\right|$	$\left|\frac{M_{1}}{\pi R^{2}}\right|$	$\left|\frac{M_{2}}{\pi R^{2}}\right|$	$\left|\frac{h_{s,1}}{\pi R^{2}}\right|$	$\left|\frac{h_{s,2}}{\pi R^{2}}\right|$	$\left|\frac{M_{2}}{\pi R^{2}}-\frac{D_{2}}{D}h_{2}\right|$	$\left|\frac{D_{2}}{D}h_{1}-\frac{M_{1}}{\pi R^{2}}\right|$	$\left|\frac{D_{2}}{D}h_{s,1}\right|$	$\left|\frac{D_{2}}{D}h_{s,2}\right|$	$\left|\frac{2D_{2}}{R}\right|$
1	4,44	4,59	1,03	10,56	13,93	0,45	0,68	6,10	4,07	2,47	3,71	1,58
2	3,72	3,80	1,02	10,39	14,00	0,45	0,68	6,25	4,17	2,45	3,67	1,57
3	2,63	2,68	1,02	10,37	14,01	0,45	0,68	6,05	4,15	2,45	3,57	1,57
4	3,84	3,94	1,03	10,50	14,11	0,45	0,68	6,18	4,11	2,47	3,71	1,58
5	3,84	3,90	1,02	10,45	14,05	0,45	0,68	6,20	4,13	2,45	3,67	1,57
6	4,08	4,33	1,06	10,47	13,90	0,45	0,68	5,84	3,90	2,54	3,82	1,63

Avg.	3,76	3,87	1,03	10,46	14,00	0,45	0,68	6,10	4,09	2,47	3,69	1,58

**Table 4:** 

	Exp.											
	1		2		3		4		5		6	
	*K*_1_	*K*_2_	*K*_1_	*K*_2_	*K*_1_	*K*_2_	*K*_1_	*K*_2_	*K*_1_	*K*_2_	*K*_1_	*K*_2_
*M* (g)	56,07	73,97	55,17	74,33	55,09	74,39	55,74	74,95	55,51	74,58	55,57	73,83
*ρ*_*s*_ (g/cm^3^)	1,69		1,73		1,72		1,74		1,72		1,63	
*ρ* = *ρ*′′ (g/cm^3^)	1,03		1,02		1,02		1,02		1,02		1,06	
*ρ*′ (g/cm^3^)	2,21		2,29		2,27		2,3		2,27		2,07	
*P*′ (%) dry matter	28,11	31,97	29,73	32,96	29,39	31,74	29,42	32,84	29,17	32,56	26,56	30,00
*P*′′ (%) bound dispersion medium in the sediment	10,29	11,69	10,37	11,53	10,38	11,21	10,26	11,43	10,30	11,49	10,69	12,07
*P* (%) free dispersion medium	61,46	56,20	59,85	55,35	59,94	56,75	60,21	55,61	60,46	55,89	62,75	57,90
